# Uncovering Genomic Causes of Co-Morbidity in Epilepsy: Gene-Driven Phenotypic Characterization of Rare Microdeletions

**DOI:** 10.1371/journal.pone.0023182

**Published:** 2011-08-17

**Authors:** Dalia Kasperavičiūtė, Claudia B. Catarino, Krishna Chinthapalli, Lisa M. S. Clayton, Maria Thom, Lillian Martinian, Hannah Cohen, Shazia Adalat, Detlef Bockenhauer, Simon A. Pope, Nicholas Lench, Martin Koltzenburg, John S. Duncan, Peter Hammond, Raoul C. M. Hennekam, John M. Land, Sanjay M. Sisodiya

**Affiliations:** 1 Department of Clinical and Experimental Epilepsy, University College London Institute of Neurology, London, United Kingdom; 2 National Society for Epilepsy, Chalfont-St-Peter, Bucks, United Kingdom; 3 Department of Haematology, University College London and University College London Hospitals NHS Foundation Trust, London, United Kingdom; 4 Renal Unit, University College London Institute of Child Health and Great Ormond Street Hospital NHS Trust, London, United Kingdom; 5 Neurometabolic Unit, National Hospital for Neurology and Neurosurgery, London, United Kingdom; 6 North East Thames Regional Genetics Service, Great Ormond Street Hospital for Children, London, United Kingdom; 7 Sobell Department of Motor Neuroscience and Movement Disorders, University College London Institute of Neurology, London, United Kingdom; 8 MRC Centre for Neuromuscular Diseases, National Hospital for Neurology and Neurosurgery, London, United Kingdom; 9 Molecular Medicine Unit, University College London Institute of Child Health, London, United Kingdom; 10 Department of Pediatrics, Academic Medical Center, University of Amsterdam, Amsterdam, The Netherlands; Instituto de Ciencia de Materiales de Madrid - Instituto de Biomedicina de Valencia, Spain

## Abstract

**Background:**

Patients with epilepsy often suffer from other important conditions. The existence of such co-morbidities is frequently not recognized and their relationship with epilepsy usually remains unexplained.

**Methodology/Principal Findings:**

We describe three patients with common, sporadic, non-syndromic epilepsies in whom large genomic microdeletions were found during a study of genetic susceptibility to epilepsy. We performed detailed gene-driven clinical investigations in each patient. Disruption of the function of genes in the deleted regions can explain co-morbidities in these patients.

**Conclusions/Significance:**

Co-morbidities in patients with epilepsy can be part of a genomic abnormality even in the absence of (known) congenital malformations or intellectual disabilities. Gene-driven phenotype examination can also reveal clinically significant unsuspected condition.

## Introduction

Epilepsy is a common neurological condition. Many patients with epilepsy have additional conditions [1]. When epilepsy is considered the index condition, co-existing conditions are considered ‘co-morbidities’. Co-morbidities degrade the quality of life in individuals with epilepsy [2]. Theoretically, co-morbidities could be causal, resultant, coincidental or have shared causation, either genetic, environmental or both. Psychiatric co-morbidities are usually attributed to seizures, drugs and shared risk factors, such as brain disease predisposing to both conditions [3]. Somatic co-morbidities are less well explored. Studying co-morbidity is important to understand basic pathophysiology and for overall clinical management. Treating co-morbidities can reduce the severity of the seizure disorder [4]. Treatment of seizures can reduce the severity of co-morbidities. Drugs used to treat one can have effects on the other [5].

Recently, chromosomal microdeletions have been implicated causing or contributing to some sporadic, ‘common’ epilepsies, both idiopathic generalized [6]–[8] and partial [9]. Epilepsy-related microdeletions often encompass multiple genes, which can generate contiguous gene syndromes. Due to variable expressivity, such genomic imbalances may not have an easily recognizable set of symptoms and signs and may not be suspected during routine clinical examination. Genetic testing is usually not considered for patients with sporadic epilepsies in the absence of manifestations pointing to a syndromic diagnosis.

Here, we present three patients with sporadic epilepsies and some recognized co-morbidities, who were each found to have a large microdeletion (≥1.4 Mb). By exploring the clinical consequences of the genetic imbalance, we show both that known co-morbidities can at least partly be explained by the microdeletion and that important unsuspected co-morbidities can be revealed, with clinical consequences for patients in both scenarios. Whilst such phenomena are familiar to many clinical geneticists, they are much less well-recognised in general to physicians caring for individuals with epilepsy.

## Materials and Methods

The study was approved by the National Hospital for Neurology and Neurosurgery and Institute of Neurology Joint Research Ethics Committee. The patients in this manuscript have given written informed consent (as outlined in the PLoS consent form) to publication of their case details.

During a multi-centre study of genetic susceptibility to epilepsy [9], genome-wide typing of single nucleotide polymorphisms had shown a putatively-pathogenic microdeletion in the three patients studied here. The patients were selected for investigation from a pool of ten on the basis of the genes known to be deleted: this was not intended to be a systematic survey of co-morbidity in patients with presumptively pathogenic microdeletions. The deletions were confirmed by array CGH (Nimblegen 135 K v3.1) in all cases, and for Case 1 also by quantitative PCR (Table 1 and Figure S1). The deletion regions were annotated in UCSC genome browser (http://genome.ucsc.edu/).

**Table 1:** Deletion regions and genes in the deleted regions.

Case	Deleted region detected using Illumina 610quad microarrays, bp*	Deleted region detected using Nimblegen 135 K v3.1microarrays, bp*	Genes in the deleted region
Case 1	Chr3:94,994,003–99,339,023	Chr3: 95,069,595–99,333,580	*PROS1, ARL13B, STX19, DHFRL1, NSUN3, LOC255025, EPHA6, ARL6, CRYBG3, MINA, GABRR3, OR5AC2, OR5H1*
Case 2	Chr12:4,419,280–5,999,525	Chr12: 4,408,710–5,978,830	*AKAP3, C12orf4, DYRK4, FGF6, GALNT8, KCNA1, KCNA5, KCNA6, NDUFA9, NTF3, RAD51AP1, TMEM16B (ANO2), VWF*
Case 3	Chr17:31,922,987–33,333,394	Chr17:31,487,760–33,574,650	*ZNHIT3, MYO19, PIGW, GGNBP2, DHRS11, MRM1, LHX1, AATF, ACACA, C17orf78, TADA2L, DUSP14, AP1GBP1, DDX52, HNF1B, LOC284100*

* NCBI build 36, Chr = chromosome.

Detailed phenotypic re-evaluation and clinical investigations were subsequently undertaken. To assess the potential consequences of each microdeletion, we performed tests based on information about the function of selected genes in the deleted region and their known involvement in various diseases other than epilepsy (see Results for each case and Text S1).

## Results

### Clinical histories

#### Case 1

This female patient aged 26 was born at 41 weeks after an uneventful pregnancy. There was no family history of seizures. At day 3, she was admitted to special care with vomiting and a single seizure was recorded. Seizures, described then as ‘asymmetric spasms’, resumed at 6 months and were treated with antiepileptic drugs. Brain X-ray computerized tomography showed a left middle cerebral artery territory ischaemic infarction, considered cryptogenic and to have occurred in the first week of life. She showed early left hand preference. Development was normal. At 3.5 years, antiepileptic drugs were tapered. At age 9, she developed complex partial seizures with right arm posturing and jerking; drugs were recommenced. Aged 10, startle seizures began, with secondarily generalized tonic-clonic seizures from age 16. Her seizures were resistant to antiepileptic drugs. Cognition remained normal.

Neurological examination at 18 years showed right hemiparesis, with good right hand function, a mild hemiparetic gait and a mild fixed right ankle deformity. No unusual morphology was noted. Cranial MRI showed the left medial cerebral artery territory infarct and left cerebral hemiatrophy. Video-EEG telemetry recorded left central interictal epileptiform discharges; semiology pointed to left hemispheric onset, compatible with the MRI lesion. The medical history was otherwise non-contributory. Seizures continue despite polytherapy. She was considered to have a common, sporadic focal epilepsy due to an early cerebral infarction. A contrast bubble study showed that the heart was structurally normal with no patent foramen ovale.

#### Case 2

This female patient aged 44 was born at term as one of twins. Her twin brother has autism and a sister has epilepsy attributed to cerebral hypoxia after accidental strangulation. There was no other family history of epilepsy. She had normal motor and cognitive development. At 19 years she had a nocturnal convulsive seizure. At 20, she developed brief complex partial seizures, starting with a cephalic aura followed by staring and cessation of activity. At 38 years, she developed left upper eyelid myokymia, considered incidental at the time. Later progression of the myokymia to intermittent facial and hand involvement was not reported by the patient, and only elicited on direct enquiry after genetic testing.

Neurological examination showed no abnormalities other than the myokymia. Interictal EEG showed occasional irregular slow waves over the left temporal area. On hyperventilation, irregular theta and delta waves appeared over the left temporal and left frontal areas. Cranial MRI showed decreased volume of the left hippocampus. Neuropsychometry showed average intellectual functioning without memory or language impairment. She has remained completely seizure-free for several years on antiepileptic drug dual therapy. She was considered to have drug-responsive, common mesial temporal lobe epilepsy due to hippocampal sclerosis, with incidental myokymia.

#### Case 3

This 31 year old male was born at term by normal delivery after an uneventful pregnancy. There was no family history of seizures. He walked at 24 months and his parents considered his development comparable to his siblings. At 30 months, he had an acute neurological illness diagnosed at the time as viral encephalitis, heralded by generalised tonic-clonic status epilepticus following an unremarkable upper airway infection. He suffered in-hospital respiratory arrest, was promptly resuscitated, and transferred to a regional neurological centre. Cerebrospinal fluid analysis showed normal protein and glucose, no red cells and three lymphocytes/mm^3^; no organism was isolated. He remained in focal status epilepticus for more than 48 hours, eventually controlled on phenobarbitone and phenytoin. He had a transient left hemiparesis. Antiepileptic drugs were tapered over three months. He remained seizure-free for seven years, but was noted to have learning difficulties. Health was otherwise excellent.

Habitual seizures started at 10 years. Seizures were never controlled despite several antiepileptic drug regimes. Seizures, occurring 2–3/week, started with a brief autonomic aura, progressing to unresponsiveness, with staring and oro-manual automatisms. Cranial MRI showed right hippocampal sclerosis (Figure S2). On EEG-videotelemetry, four habitual partial seizures were recorded; lateralising semiological signs included nose rubbing with his right hand and post-ictal urinary urge; ictal EEG showed bitemporal rhythmic theta discharge, more prominent and sustained on the right, with right temporal interictal epileptiform discharges. Neuropsychometry showed mild learning disability, globally weak memory, but low risk of global amnesia in the context of left temporal structural normality. At 28, he had a right anterior temporal lobectomy. He has been free of seizures to the more recent follow-up at 36 months, with tapering of antiepileptic drugs. Repeat neuropsychometric assessment showed improvement of attention span, verbal memory and spatial reasoning skills; his visual memory and higher-level visual processing skills had slightly declined. Histopathology of the lobectomy specimen confirmed hippocampal sclerosis, without mossy fibre sprouting (Figure S2). He was considered to have surgically-remediable, typical sporadic mesial temporal lobe epilepsy due to hippocampal sclerosis. The medical history was otherwise unremarkable.

### Gene-Driven Clinical Investigations

#### Case 1 (del 3q11.2)

The microdeletion included *PROS1* (Table 1), which encodes protein S, a vitamin K-dependent plasma protein co-factor for the anticoagulant protease activated protein C, that itself inhibits activated factors V and VIII. *PROS1* mutations and deletions cause autosomal dominant hereditary thrombophilia [10]. Given the unexplained early cerebral infarction, we suspected protein S functional deficiency, since thrombophilia is a stroke risk factor in neonates and children [11]. Case 1 had free and functional protein S deficiency (respectively 0.31 IU/ml, normal range 0.50–1.34; 0.28 IU/ml, normal range 0.50–1.20), confirmed on repeat testing four months later. She was heterozygous for the C677T *MTHFR* polymorphism, with normal total serum homocysteine. The remainder of the thrombophilia screen, including antiphospholipid antibodies, which may induce acquired protein S deficiency, yielded normal results. Management consequences resulting from these findings comprised advice on risk reduction measures against thrombosis, including maintenance of hydration, avoidance of oestrogen-containing hormonal contraception, and low molecular-weight heparin thromboprophylaxis in situations with increased thrombotic risk. Long-term prophylactic anticoagulation was not indicated.

On directed questioning after these findings, a family history of thrombophilia emerged. Her maternal grandfather had had three known pulmonary emboli, from the age of 30 years, and had been anticoagulated until death at 56. Both aCGH and quantitative PCR analysis revealed that her mother also had the same *PROS1* deletion (Figure S1 for quantitative PCR results). She has low free protein S (0.39 IU/ml) and normal functional protein S (0.59 IU/ml), is healthy and has never had thrombotic events or pregnancy losses. Such variable expressivity of *PROS1* deletions is well known [10].

Dense surface modelling analysis of the patient's face did not uncover facial dysmorphism (Figure S4, Video S1).

#### Case 2 (del 12p13.32-p13.31)

**Figure 1:**
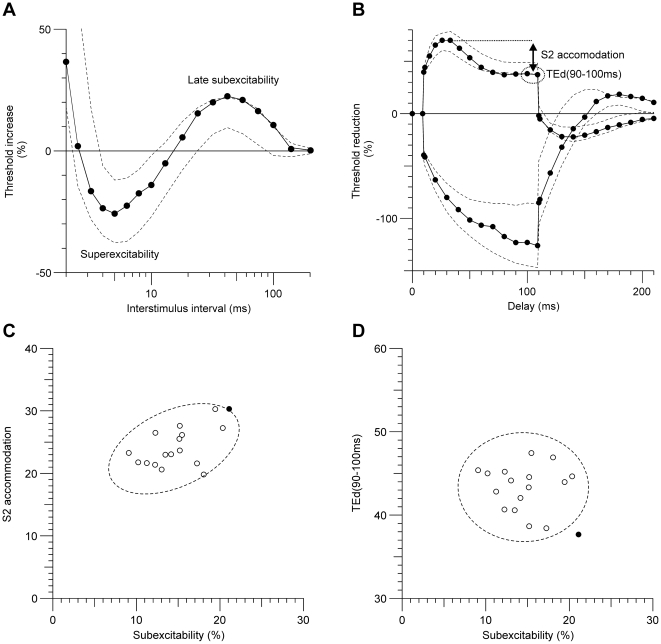
Nerve excitability profile of Case 2. In the nerve excitability profile, there was an abnormally enhanced late subexcitability of the recovery cycle (**A**) and a pronounced S2 accommodation and reduced TEd (90–100 ms) value at the end of the depolarising threshold electrotonus (upper trace in **B**). **C,D**: scatter plots of these nerve excitability parameters in the patient (filled circle) and 16 normal controls (open symbols). Dashed lines (A–D) show 95% confidence interval of the normal range. See Text S1 for detailed description of methods.

The microdeletion included *KCNA1*, *KCNA5* and *VWF* genes (Table 1).*KCNA1* encodes a voltage-gated delayed potassium channel (K_v_1.1). *KCNA1* mutations cause episodic ataxia type 1 with myokymia (EA1). Nerve excitability testing in the patient showed several abnormalities (Figure 1) which are also observed in patients with EA1 [12]. However, the patient was on carbamazepine at the time of testing and therefore blockade of voltage-gated sodium channels may have contributed to some of the observed changes, which may not be solely due to reduced K_v_1.1 function. During needle electromyography the patient did not have fine involuntary finger movements and testing showed normal motor unit discharges despite obvious myokymia during other observations.

*KCNA5* encodes a voltage-gated potassium channel (K_v_1.5) expressed in human atria. *KCNA5* loss-of-function mutations cause atrial fibrillation [13]. Case 2 had had unprovoked, brief, paroxysmal episodes of fast palpitations, without other symptoms. On directed questioning, it emerged that her father (deceased) had had paroxysmal atrial fibrillation from his forties, and had been on treatment. The patient's resting electrocardiogram and 24-hour ambulatory electrocardiogram, both performed when she was asymptomatic, were normal. Further ECG monitoring is planned.

*VWF* encodes von Willebrand factor (vWF) glycoprotein that binds platelets via glycoprotein 1b/IX to subendothelial collagen and carries factor VIII. vWF is crucial to haemostasis. *VWF* mutations cause von Willebrand disease, but incomplete penetrance is known [14]. Factor VIII (1.4 IU/ml, normal range 0.5–2.0), vWF antigen (1.24 IU/ml, normal range 0.5–1.6) and ristocetin cofactor (1.04 IU/ml, normal range 0.5–2.0) levels were all normal.

Dense surface modelling analysis of the patient's face did not uncover facial dysmorphism (Figure S4, Video S2).

#### Case 3(del 17q12)

The deletion encompasses *ACACA* and *HNF1B* (Table 1). *HNF1B* mutations and deletions cause renal cyst and diabetes syndrome [15] and hypomagnesemia [16]. The subject's oral glucose tolerance test (OGTT) showed a normal glucose profile, but in keeping with the *HNF1B* deletion, the dynamic insulin response was impaired: at 60 minutes, with peak glucose concentration, his insulin level was >8 standard deviations below the published mean (Figure S3 for dynamic profiles of glucose, insulin, and other metabolites). His HbA1c level, representing an integrated measure of glycemic control over the preceding 120 days, was 5.9%, at the upper limit of the normal range (4.0–6.0%). Serum magnesium was low (0.54 mmol/L, normal range 0.6–1.0 mmol/L). Renal ultrasound showed bilateral cysts (not shown). Estimated glomerular filtration rate was low (84 mL/min/1.73 m^2^), indicating mildly reduced kidney function (chronic kidney disease stage 2, www.renal.org/CKDguide/ckd.html). These findings fit with the known consequences of *HNF1B* deletion, but none were suspected or detected before discovery of the microdeletion.

The metabolic profile for this patient is complex. *ACACA* encodes acetyl co-A carboxylase 1 (ACC1), the key enzyme in hepatic fatty acid synthesis [17]. We hypothesized that the *ACACA* deletion might cause lower levels of ACC1, altering fatty acid metabolism. Fasting total triglyceride levels were low (0.4 mmol/L, normal range 0.4–2.3 mmol/L). Acetyl-CoA carboxylation to malonyl-CoA cannot directly be measured in blood. However, its impairment could elevate citrate levels. Citrate is normally undetectable in urine. His fasting urine had no detectable citrate, but a sample after OGTT showed citraturia, compatible with hypercitratemia (not measured). This suggests impaired citrate breakdown by ATP-citrate lyase, the enzyme responsible for this conversion, which may be overwhelmed by mass action from excess citrate production, as well as being impaired by a lack of insulin activation. The alternative interpretation of an increased flux through the citrate synthetic (tricarboxylic acid cycle) pathway is unlikely, because his mitochondrial beta-oxidation is normal at rest and normally-suppressed through the OGTT (as reflected by dynamic total and fractional acyl-carnitine levels, Figure S3).

These observations are consistent with deficient ACC1 activity and with lack of dynamic activation of ACC1 in the context of an impaired insulin response. The absence of crotonyl derivatives on gas chromatography mass spectroscopy (data not shown) excludes a generalised functional biotin co-enzyme deficiency, which might have been an alternative explanation.

**Figure 2:**
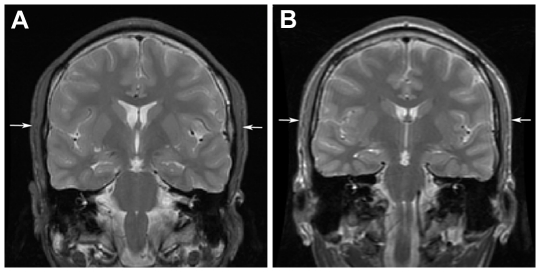
Attenuation of subcutaneous fat in Case 3. (**A**) Coronal T2-weighted image showing the patient has attenuation of scalp subcutaneous fat (arrows). (**B**) Normal appearance in a patient with epilepsy without 17q12 deletion (arrows). Subcutaneous scalp fat was present on cranial MRI in 150 patients with temporal lobe epilepsy who did not carry this deletion.

Consistent with disordered fat metabolism, his body build was lean (body mass index (BMI) 17.5 kg/m^2^, 2^nd^ centile WHO-CDC scale). Re-inspection of his cranial MRI showed attenuation of subcutaneous fat deposits (Figure 2). *ACACA* and *ACLY* (the gene encoding ATP-citrate lyase) are both known to be involved in lipogenesis in human adipose tissue and are regulated in a complex fashion by body energetics [18], though we have not uncovered the precise mechanism in our patient. We note also that in a paediatric cohort of 11 patients with renal cysts sharing the same deletion [16], wide variation of BMI was observed (2nd–100th centile, median 67th centile), suggesting overall that variable expressivity and other factors contribute to BMI, which is a blunt phenotype. His serum levels of fat-soluble vitamins A and E were normal. His low serum vitamin D level is difficult to interpret, possibly attributable to chronic therapy with enzyme-inducing antiepileptic drugs.

**Figure 3:**
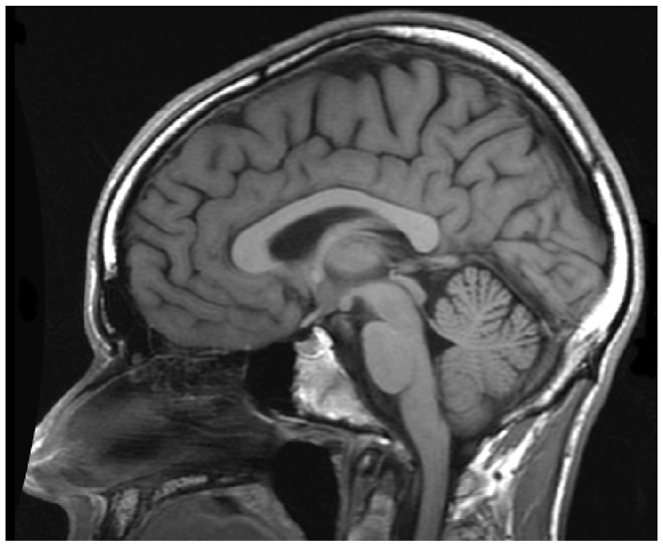
Cerebellar atrophy in Case 3. T1-weighted MRI reveals cerebellar atrophy. Note this is a T1-weighted image (not a T2-weighted image as in Figure 2A); the bright layer in the scalp is diploe, not subcutaneous fat.

Other predicted complex downstream consequences of deficient ACC1 function include ubiquinone deficiency, found to be present in the patient (33 pmol/mg; normal range 37–133 [19]). Ubiquinone deficiency is a cause of cerebellar ataxia, cerebellar atrophy or hypoplasia [20]. The patient's father noted that the patient had always lacked dexterity and agility, being able as a child, for example, to run a sprint but not hurdles. His cranial MRI aged 28 showed mild cerebellar atrophy (Figure 3). These findings are all in keeping with functional deficiency in ACC1, although an additional contribution from LHX1 deficiency cannot be excluded for the cerebellar phenotype [21]. Deficits in coordination and motor skills in patients with chromosome 17q12 deletion have been reported recently [22].

**Figure 4:**
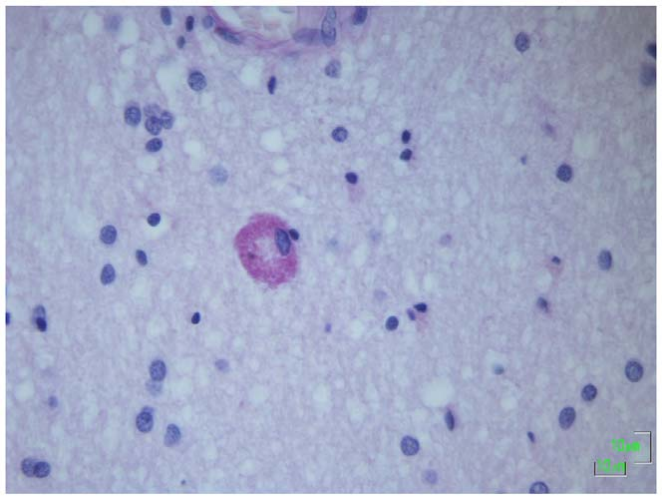
Neuropathology of resection specimen from Case 3. In the deep white (periventricular region) of the temporal lobectomy specimen of Case 3, a single cluster of globoid cells with granular cytoplasm was present. The cells showed PAS positivity, which could indicate glycogen storage, but the cells did not contain myelin debris and there was no evidence of demyelination. The cells were only present focally. Further investigation regarding histogenesis of these cells and characterization of cytoplasmic contents was not possible. Similar cell types were not noted in the hippocampus.

**Figure 5:**
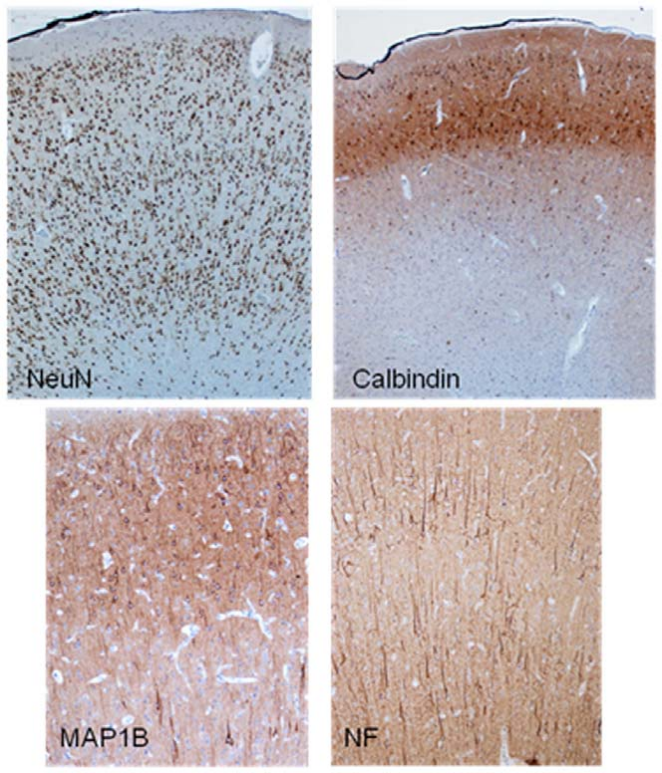
Cortical lamination in Case 3. Examination with several cortical layer markers (antibodies against proteins denoted in each panel) revealed normal cortical lamination in Case 3.

The *LHX1* gene, also residing in the deleted region, is a homeobox gene expressed in the developing brain and involved in neuronal differentiation [23] and cortical lamination [24]. In mouse models, the *Lhx1* gene is required for regulating the vertebrate head organizer [25]. Therefore, *LHX1* emerges as a plausible candidate responsible for the neurological phenotypes in patients with chromosome 17q12 deletion [22], possibly affecting brain development. Review of the tissue resected at temporal lobectomy of Case 3 showed a few cells deep in the temporal lobe white matter that contained abnormal storage material, that was PAS-positive, suggestive of excessive glycosylation (Figure 4). The nature of the material could not be further clarified. No such changes have been noted in over 200 other temporal lobectomy specimens resected from patients with drug-resistant mesial temporal lobe epilepsy [26]. However, cortical lamination, which we considered might have been affected by LHX1 deficiency, was normal when examined with several cortical layer markers (Figure 5). Neither have we detected any retinal abnormalities (Text S1).

**Figure 6:**
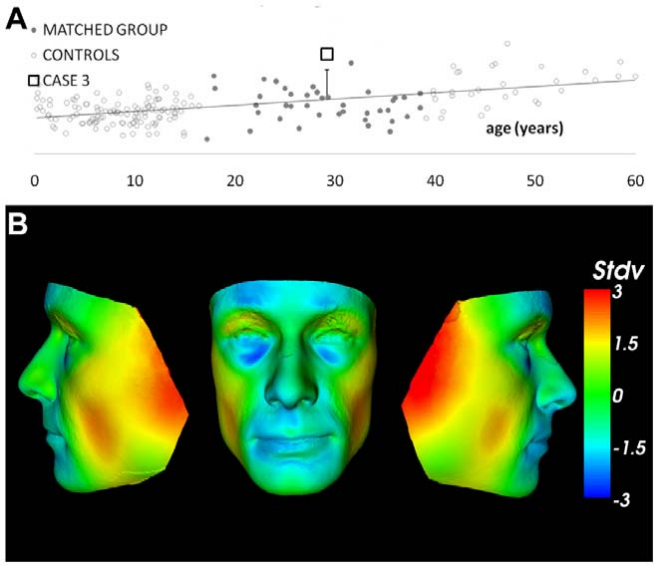
Facial dysmorphism in Case 3. (**A**) Dense surface morphology (DSM) results (See Text S1 for details). The scatter plot shows age (horizontally) against DSM distance (vertically) between the matched mean face and the patient and 200 control faces. Distance from the patient-matched mean face was linearly regressed against age for all controls. The patient was fitted to the appropriate regression and a 95% confidence interval was calculated for the predicted distance from the patient-matched mean. The scatter plot shows that Case 3 (square) is well outside the 95% confidence interval (bar). (**B**) Heat maps using a red–green-blue spectrum to depict inward–null-outward displacement along the surface normal for the patient face relative to its matched mean face using 50 control subjects. Displacement is expressed as the number of standard deviations (Stdv) from the mean. The lateral patches of red and yellow reflect an unusually narrow face. The red and yellow cheek patches and the null displaced green islands in the gonial region of the lower jaw reflect diminished fatty tissue rather than reduced gonial/skeletal width. Some fullness of the perioral region is shown (blue).

Clinical genetic examination after discovery of the microdeletion showed subtle evidence of dysmorphism, not articulated by several neurologists during pre-surgical evaluation: he had a narrow face, mildly receding forehead, little facial subcutaneous fat, prominent nasal bridge, broad nasal base, long and wide philtrum and large mouth. His shoulders were narrow, and he had pectus excavatum, limited elbow extension, flat hands, prominent finger joints, mild cutaneous 2–3 finger syndactyly, clinodactyly of the 5th fingers, under-developed hypothenar regions and narrow feet. Facial dysmorphism was confirmed by quantitative analysis in comparison with 200 age-, sex- and ethnicity-matched controls (Figure 6, Video S3). It should be emphasised that on first examination at our centre, facial abnormalities were not identified, and these were only documented by formal clinical genetics review and confirmed by quantitative analysis after discovery of the microdeletion.

These manifestations are not described in most other published cases with overlapping 17q12 deletions, including 14 patients with autism, schizophrenia or seizures [22], [27], [28]. However, several facial photographs of cases in the report by Moreno-De-Luca *et al.*, 2010 [22], especially case 1, show overlapping facial features. These include a narrow face, broad nasal base and large mouth. This comparison indicates the difficulties in describing the human face and the importance of having faces evaluated by specialist dysmorphologists, or through the use of objective techniques such as dense surface modelling.

None of the findings above were associated with any symptoms volunteered by the patient, nor revealed during any investigations prior to specific phenotypic re-evaluation after discovery of the microdeletion. Some of the findings have direct clinical consequences for the patient: he has been started on magnesium and ubiquinone supplements and will undergo regular monitoring of renal function and glucose levels.

## Discussion

We describe three patients with epilepsy, in whom co-morbidities are explicable by the same microdeletion that is putatively related to each individual's epilepsy itself, consistent with the genomic disease model. Notably, the microdeletions were identified in adults who were considered before the study to have non-syndromic epilepsies associated with underlying cerebral abnormalities (cerebral infarction, hippocampal sclerosis). None were known to have congenital anomalies or intellectual disabilities. Extended phenotypes were sought only after discovery of microdeletions. Previously, the co-morbidities had either been considered unrelated to the epilepsy (e.g. myokymia) or not suspected at all (e.g. thrombophilia, metabolic derangement and kidney dysfunction). In all three patients, the findings had clinical consequences for counselling, further clinical investigation or monitoring, and additional treatment regarding the co-morbidity.

We emphasise this study is not a systematic survey. We selected 3/10 patients from one centre from our original multi-centre study [9] on the basis that their microdeletions contained genes that might cause explorable co-morbidities, in order to illustrate the concept of epilepsy as one manifestation of a genomic condition. A systematic search for underlying genetic causes of epilepsies with co-morbidities would seem warranted.

The individual details are of interest. In Case 1, we postulate that *PROS1* deletion-related thrombophilia predisposed to cerebral infarction, the location of which was compatible with causation of the patient's epilepsy. In Case 2, myokymia was initially considered to be unrelated to the epilepsy, but both might be explained by deletion of *KCNA1*. Effects of *KCNA1* mutations, at least in some cases, are attributable to a dominant-negative mechanism [29]; the mechanism in Case 2, with a microdeletion, is not yet known. In Case 3, *ACACA* deletion is associated with disordered endogenous fatty acid metabolism. This may cause the attenuated subcutaneous fat seen in our patient. Loss of one copy of the gene seems to have modest effect on the overall health of the patient. It is possible that the infantile ‘encephalitic’ illness represented ACC1 deficiency-related metabolic crisis, causing hippocampal sclerosis which in turn led to epilepsy. Modulation of acetyl-coenzyme A carboxylases ACC1 and ACC2 is being proposed as a treatment for obesity [30], but this case's information may indicate it not be a harmless option, at least in childhood.

Determining the pathogenicity of a given microdeletion presents a challenge shared across diseases: several methods have been suggested [31], but none is necessarily absolute in itself. Establishing functional consequences as described here is one method of determining clinical significance; the microdeletions in this report are clinically significant for at least the co-morbidities in these patients. In our original report, microdeletions >1 Mb in size were significantly associated with epilepsy. None of the microdeletions reported here were found in 1299 controls [9]; therefore apart from the speculative mechanisms explaining co-morbidities considered above, these microdeletions might also contain other candidate genes for epilepsy. Although in Case 1, and likely in Case 2, the microdeletion was inherited from a parent without known epilepsy, the microdeletion may still be a risk factor for the epilepsy in the proband: microdeletions with variable phenotypic expressivity are commonly inherited from unaffected parents, as shown both in epilepsy [7] and other neuropsychiatric conditions [32]. Many smaller microdeletions have been similarly implicated in a variety of epilepsies [33].

With the increased use of genomic microarrays both in research studies and clinical practice, more and more clinically relevant copy number variants are being detected. Broadly, they fall into two classes – (i) highly penetrant with clear, recurrent clinical associations and (ii) those with variable expressivity found in individuals with a broad range of phenotypes [32], [34]. The mechanism of action of most of these copy number variants is unknown. Our results suggest that clinical investigations based on the knowledge of genes in the region of a copy number variant may provide insights into the mechanism of disruption caused by that copy number variant. For rare or private (i.e. non-recurrent) copy number changes, clinical testing driven by genetic hints, as in our Case 1 with loss of *PROS1*, can be informative and might confirm that a given microdeletion is functionally important, and establish a likely genotype-phenotype association. Moreover, such gene-driven investigations may reveal novel, unappreciated phenotype correlations even in already well-described syndromes with well-established clinical consequences, as shown here for the 17q12 microdeletion.

It should be noted that we had no reason to study these genes in these patients *a priori*, because (a) the originally-documented epilepsy phenotypes of the patients were not unusual for their respective ‘common’ epilepsies, (b) there were no obvious indications for cytogenetic testing (before assessment by a clinical geneticist): co-morbidities were present but there was neither intellectual disability nor obvious congenital anomalies; and (c) there was no specific clue in the phenotype for a particular locus. Detecting significant microdeletions can thus provide additional insights to human biology at an individual level. This concept itself is not novel, being well-appreciated by clinical geneticists, and reported in obviously syndromic epilepsies, but the present findings suggest that the concept also applies to apparently non-syndromic, sporadic epilepsies – which make up the vast majority of cases of epilepsy. Non-syndromic epilepsies can thus be part of unsuspected complex conditions. The converse is likely to apply as well: some patients with, for example, thrombophilia, may be known to have epilepsy as a co-morbidity, and this may in fact represent an unsuspected genomic abnormality. Wider appreciation of genomic abnormalities linking co-morbidities may bring clinical specialties back to closer daily interaction, especially as it is known that specialists often overlook co-morbidities [35], [36].

With the personal genome ‘on request’ on the horizon through next generation sequencing, detailed phenotyping and clinical investigations will be a critical complement and allow interpretation of genomic findings, dissection of the core phenotype, and promotion of health and disease prevention [37]. There are intricacies and ethical issues to be surmounted [38], and in each case the work will be time-consuming, but there are also significant potential benefits for clinical practice and science, and especially for improved and comprehensive health care for individual patients.

## Supporting Information

Figure S1 ***PROS1*** **copy number analysis by quantitative PCR.** Results of *PROS1* copy number analysis by quantitative PCR analysis. RQ -relative quantity.
(TIF)

Figure S2 **Hippocampal sclerosis in Case 3.** (**A**) T1-weighted magnetic resonance imaging of the Case 3 revealed right hippocampal volume loss (arrow) compatible with hippocampal sclerosis. (**B**) Histopathology of the lobectomy specimen from Case 3 confirmed hippocampal sclerosis, without mossy fibre sprouting. There was neuronal loss in CA1, CA4 and gliosis on GFAP. The section shown is immunolabelled for NeuN.
(TIF)

Figure S3 **Dynamic profiles of glucose, insulin, and other carnitine esters.** Dynamic profiles of glucose, insulin, and other carnitine esters. NEFA - non-esterified fatty acids.NEFA - non-esterified fatty acids.
(TIF)

Figure S4 **Dense surface morphology (DSM) results for Case 1 and Case 2.** (**A**) The scatter plot shows age (horizontally) against DSM distance (vertically) between the matched mean face and the patient and 200 control faces. Distance from the patient-matched mean face was linearly regressed against age for all controls. The patient was fitted to the appropriate regression and a 95% confidence interval was calculated for the predicted distance from the patient-matched mean. The two scatter plots show that Case 1 is just within the 95% confidence interval; Case 2 is close to the predicted value. (**B, C**) Heat maps using a red–green-blue spectrum to depict inward–null-outward displacement respectively along the surface normal of the case face relative to its matched mean face (of 50 control subjects). Displacement is expressed as the number of standard deviations from the mean. (**B**) For Case 1, the long and full nose is indicated by blue patches; the opposing blue-yellow patches on the alae reflect a rightward deflection of the nose. Cyan patches on the cheeks emphasise a broad zygomatic arch. Red on the forehead and periorbital regions indicate a backward slope and deeper set eyes respectively. The mild recession of the mandible results in a red chin. (**C**) For Case 2, yellow under the chin and cyan patches on the cheeks reflect a slightly shorter and squarer face than the matched mean. Some flattening of the supraorbital regions is shown red. A small degree of flattening of the nasal bridge is also shown (red).
(TIF)

Text S1 **Supplementary methods and results.**
(DOC)

Video S1 **Animations of face morphs.** Animations of face morphs offer the best visualisation of face shape differences when comparing each case and its age/sex-matched mean face. The unusual narrowness of Case 3's face is dysmorphic. S1 - Case 1.
(WMV)

Video S2 **Animations of face morphs.** Animations of face morphs offer the best visualisation of face shape differences when comparing each case and its age/sex-matched mean face. The unusual narrowness of Case 3's face is dysmorphic. S2 - Case 2.
(WMV)

Video S3 **Animations of face morphs.** Animations of face morphs offer the best visualisation of face shape differences when comparing each case and its age/sex-matched mean face. The unusual narrowness of Case 3's face is dysmorphic. S3 - Case3.
(WMV)
