# Supplementary figures and images for: Uncovering Genomic Causes of Co-Morbidity in Epilepsy: Gene-Driven Phenotypic Characterization of Rare Microdeletions
Source: PLoS One. 2011 Aug 17;6(8):e23182. doi: 10.1371/journal.pone.0023182 (PMC3157359; doi:10.1371/journal.pone.0023182)

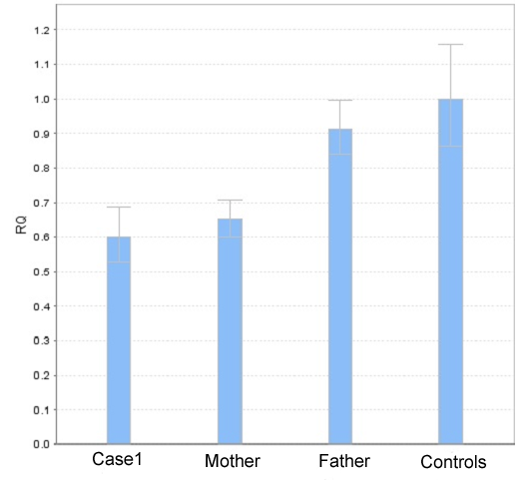

Supplement: Figure S1 — PROS1 copy number analysis by quantitative PCR. Results of PROS1 copy number analysis by quantitative PCR analysis. RQ -relative quantity. (TIF) [file pone.0023182.s001.tif]

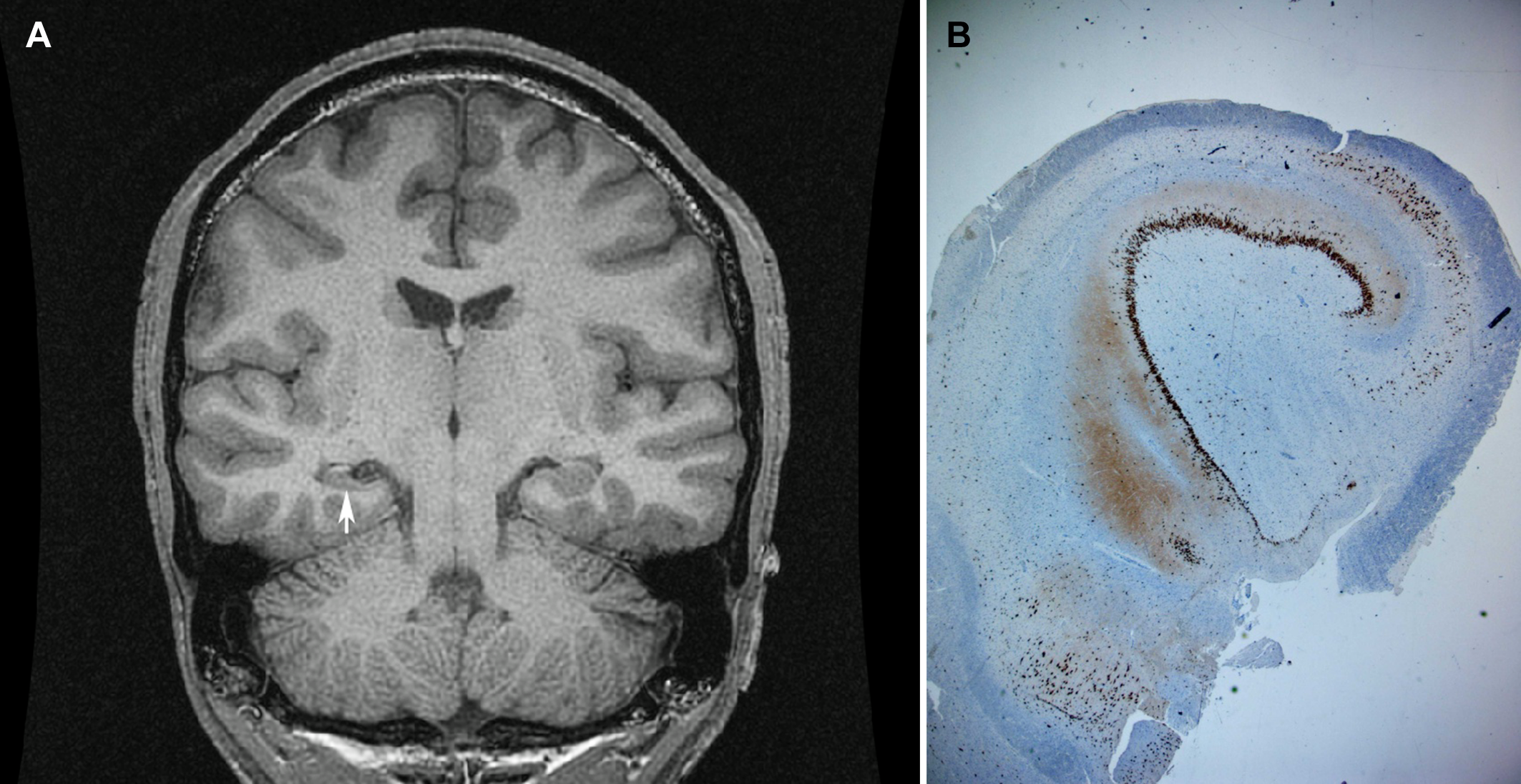

Supplement: Figure S2 — Hippocampal sclerosis in Case 3. (A) T1-weighted magnetic resonance imaging of the Case 3 revealed right hippocampal volume loss (arrow) compatible with hippocampal sclerosis. (B) Histopathology of the lobectomy specimen from Case 3 confirmed hippocampal sclerosis, without mossy fibre sprouting. There was neuronal loss in CA1, CA4 and gliosis on GFAP. The section shown is immunolabelled for NeuN. (TIF) [file pone.0023182.s002.tif]

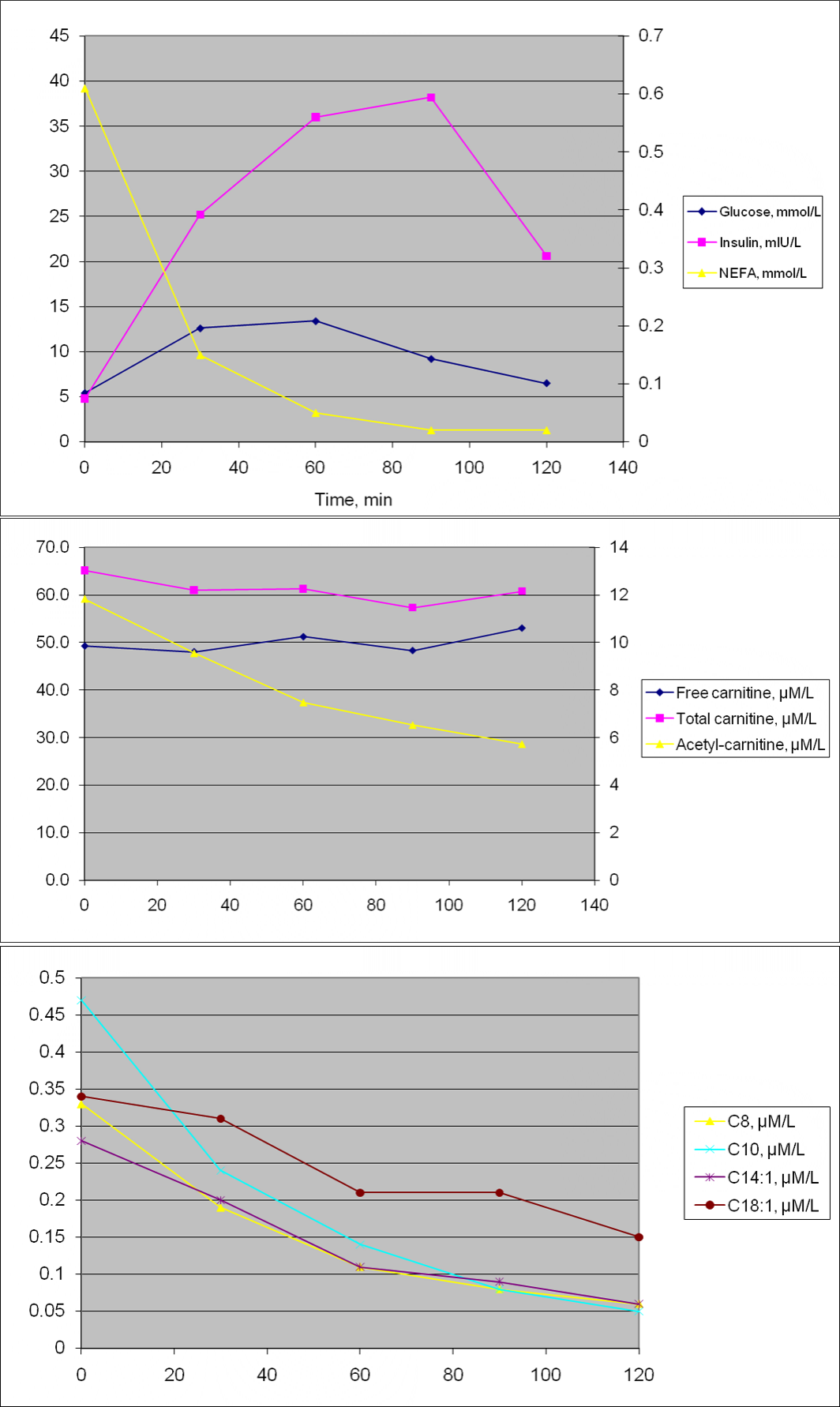

Supplement: Figure S3 — Dynamic profiles of glucose, insulin, and other carnitine esters. Dynamic profiles of glucose, insulin, and other carnitine esters. NEFA - non-esterified fatty acids.NEFA - non-esterified fatty acids. (TIF) [file pone.0023182.s003.tif]

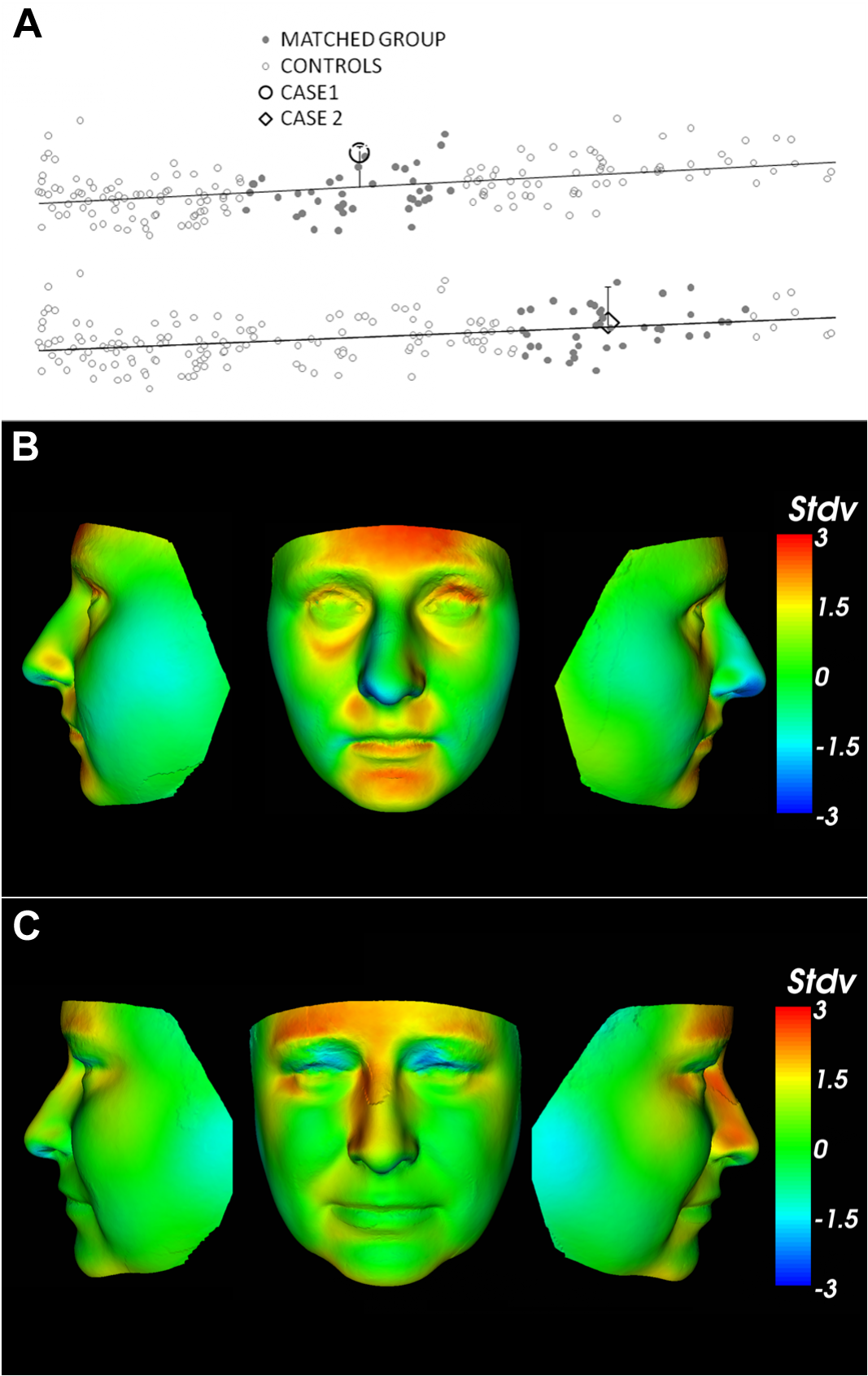

Supplement: Figure S4 — Dense surface morphology (DSM) results for Case 1 and Case 2. (A) The scatter plot shows age (horizontally) against DSM distance (vertically) between the matched mean face and the patient and 200 control faces. Distance from the patient-matched mean face was linearly regressed against age for all controls. The patient was fitted to the appropriate regression and a 95% confidence interval was calculated for the predicted distance from the patient-matched mean. The two scatter plots show that Case 1 is just within the 95% confidence interval; Case 2 is close to the predicted value. (B, C) Heat maps using a red–green-blue spectrum to depict inward–null-outward displacement respectively along the surface normal of the case face relative to its matched mean face (of 50 control subjects). Displacement is expressed as the number of standard deviations from the mean. (B) For Case 1, the long and full nose is indicated by blue patches; the opposing blue-yellow patches on the alae reflect a rightward deflection of the nose. Cyan patches on the cheeks emphasise a broad zygomatic arch. Red on the forehead and periorbital regions indicate a backward slope and deeper set eyes respectively. The mild recession of the mandible results in a red chin. (C) For Case 2, yellow under the chin and cyan patches on the cheeks reflect a slightly shorter and squarer face than the matched mean. Some flattening of the supraorbital regions is shown red. A small degree of flattening of the nasal bridge is also shown (red). (TIF) [file pone.0023182.s004.tif]
