# Supplementary material for: Uncovering Genomic Causes of Co-Morbidity in Epilepsy: Gene-Driven Phenotypic Characterization of Rare Microdeletions
Source: PLoS One. 2011 Aug 17;6(8):e23182. doi: 10.1371/journal.pone.0023182 (PMC3157359; doi:10.1371/journal.pone.0023182)
Supplement: Text S1 — Supplementary methods and results. (DOC) [file pone.0023182.s005.doc]

# Supplementary methods

**Annotation of deletions**

Information on other cases with similar deletions was retrieved from public databases -- Database of Genomics Variants [1], DECIPHER [2], CHOP CNV [3], ECARUCA [4] -- and through literature searches.

**PROS1 copy number analysis**

PROS1 gene copy number analysis was performed by real time PCR quantitative polymerase chain reaction (qPCR) using SYBR green on DNA samples from the proband, mother, father and three normal controls. A “no DNA” control was also included. qPCR primer sequences were designed using Invitrogen OligoPerfect^TM^ software and checked for SNPs using SNPCheck (National Genetics Reference Laboratory, Manchester, UK). Forward (5’CAGAAACAATCTGGAGTTGTCG3’) and reverse (5’GGCCACTTTTGCTTTCATTG3’) primers were design to a 110bp region of the PROS1 gene. PCRs were performed in a total reaction volume of 20μl containing 25ng DNA, 10μl of 2X SYBR Green Master Mix (Applied Biosystems, Life Technologies, Carlsbad, CA, USA) and 300nM of each primer and an amplification profile of 10mins at 95^o^C followed by 40 cycles of 95^o^C/1min; 60^o^C/1min. Data were normalised against two reference sequences ACTBL2 (5q11.2) and MANEA (6q16.1). All samples were run in triplicate and data analysed with the instrument (Applied Biosystems StepOnePlus) software and further analysed using the comparative ddC_t_ method [5].

**Nerve excitability tests**

A nerve excitability profile of motor axons of the median nerve was obtained as described previously [6]. The recovery cycle following a supramaximal stimulus consists of three phases: (i) initial refractoriness, followed by (ii) superexcitability and (iii) late subexcitability. It is measured following a supramaximal 0.5-ms conditioning stimulus. The excitability changes are recorded at 18 conditioning-test intervals, decreasing from 200 ms to 1.5 ms in an approximately geometric sequence. Two responses are averaged for each stimulus level.

Threshold electrotonus examines changes of the nerve excitability in response to 100-ms-long subthreshold depolarizing or hyperpolarizing currents. Threshold changes are assessed at defined delays during and after the start of the polarizing current (‘threshold electrotonus’). An unconditioned control stimulus, and the depolarizing or hyperpolarizing conditioning stimulus together with the test stimulus, are tested in turn and 3 responses are averaged for each stimulus level. The S2 accommodation describes the change of threshold from the peak of the depolarizing threshold electrotonus to its end, TEd (90-100ms).

**Dense surface morphology.**

The patients were compared to a group of matched-sex control subjects with a matching mean age. Two DSMs were computed, each containing the patient and about 200 matched-sex controls. The mean face of the age/sex-matched control group was used for face shape comparisons to detect the degree of dysmorphism in each patient. Scatter plots showing age (horizontally) against Dense surface morphology (DSM) distance (vertically) between the matched mean face and the patient and all control faces were produced. Distance from each patient-matched mean face was linearly regressed against age for all controls. Each patient was fitted to the appropriate regression and a 95% confidence interval was calculated for the predicted distance from the patient-matched mean.

# Supplementary results

**PROS1 copy number analysisby qPCR**

qPCR results are presented in Supplementary Figure S1. Using the control DNA as a reference, the father of Case 1 has a normal copy number (relative quantity (RQ) 0.91), whilst the Case1 and her mother have mean values of 0.60 and 0.65 respectively, consistent with a hemizygous deletion at the PROS1 locus.

**Exploration of possible LHX1 related phenotypes in case 3**

The mouse homolog of the *LHX1* is involved in retinal horizontal cell development [7], abnormality of which might be expected to cause non-progressive retinal dysfunction affecting the inner retina. The patient's visual fields on Goldmann perimetry were normal. His retinal nerve fiber layer was normal, with no evident abnormality of retinal layering when measured using optical coherence tomography. Electroretinography was not performed.

**Dynamic profiles of glucose, insulin, and other metabolites in Case 3**

Dynamic profiles of glucose, insulin, and other metabolites are shown in Supplementary Figure S3. Data on normal dynamic insulin response are from Stevic et al., 2007 [8] and Seljeflot et al., 1994 [9].

**Dysmorphology analysis in Case 1 and Case 2**

The dysmorphology analysis revealed mild facial dysmorphism in case 1 and no dysmorphism in case 2 (Figure S4).

## References

1. Iafrate AJ, Feuk L, Rivera MN, Listewnik ML, Donahoe PK, et al. (2004) Detection of large-scale variation in the human genome. Nat. Genet 36: 949-951.

2. Firth HV, Richards SM, Bevan AP, Clayton S, Corpas M, et al. (2009) DECIPHER: Database of Chromosomal Imbalance and Phenotype in Humans Using Ensembl Resources. Am. J. Hum. Genet 84: 524-533.

3. Shaikh TH, Gai X, Perin JC, Glessner JT, Xie H, et al. (2009) High-resolution mapping and analysis of copy number variations in the human genome: a data resource for clinical and research applications. Genome Res 19: 1682-1690.

4. Feenstra I, Fang J, Koolen DA, Siezen A, Evans C, et al. (2006) European Cytogeneticists Association Register of Unbalanced Chromosome Aberrations (ECARUCA); an online database for rare chromosome abnormalities. Eur J Med Genet 49: 279-291.

5. D’haene B, Vandesompele J, Hellemans J (2010) Accurate and objective copy number profiling using real-time quantitative PCR. Methods 50: 262-270.

6. Tomlinson S, Burke D, Hanna M, Koltzenburg M, Bostock H (2010) In vivo assessment of HCN channel current (I(h)) in human motor axons. Muscle Nerve 41: 247-256.

7. Poché RA, Kwan KM, Raven MA, Furuta Y, Reese BE, et al. (2007) Lim1 is essential for the correct laminar positioning of retinal horizontal cells. J. Neurosci 27: 14099-14107.

8. Stevic R, Zivkovic TB, Erceg P, Milosevic D, Despotovic N, et al. (2007) Oral glucose tolerance test in the assessment of glucose-tolerance in the elderly people. Age Ageing 36: 459-462.

9. Seljeflot I, Eritsland J, Torjesen P, Arnesen H (1994) Insulin and PAI-1 levels during oral glucose tolerance test in patients with coronary heart disease. Scand. J. Clin. Lab. Invest 54: 241-246.
